# Monthly Summary

**Published:** 1892-09

**Authors:** 


### Monthly Summary.

COMPOUND FILLINGS.—One of the greatest economies in operative dentistry, and the best class of fillings for large cavities, are compound fillings of alloy or oxyphosphate as the main filling, with gold as a cap; or oxyphosphate as a filling and alloy as a cap. These are not only more economical than all gold, but better. An all gold filling can not be put in as perfectly as alloy or oxyphosphate, and yet, when capped with gold, the filling is as presentable as all gold. The advantage of filling a large cavity with oxyphosphate and capping with alloy is, that if the oxyphosphate is used soft and is of good quality, it adheres to the walls of the cavity, and is, therefore, impervious, while the alloy is more enduring as a surface.

All these fillings may be allowed first to harden, and then a portion cut out for capping, or the capping may be incorporated with the filling while soft. If the latter, only a small portion of the capping material is pressed into the filling while soft, and then, as the filling sets, more is added, first next to the walls, and gradually extended to the center, and then sufficient is added to complete the filling. Or a thin plate of gold can be fitted to the shape of the cavity, and, after two or three short pins are soldered to the under side, it is pressed on to the soft filling. If you are not sure of its articulation with other teeth, first fill the cavity with wax, and try it on; if on the grinding surface, the plate should conform to the contour of the tooth.

Also, after a root has been properly prepared, a gold ferrule of thin gold plate may be made, and this capped with a plate of the same material for a grinding surface. Then fill both the skeleton tooth and the root with oxyphosphate and place in position. It makes a durable artificial tooth.—*Items of Interest.*

---

AMALGAMS.—That there is a great difference in amalgams for filling teeth is generally conceded. A recent writer treating on amalgams says: "Brittleness being one of the weaknesses of amalgams, every possible precaution should be taken to prevent thin and sharp edges." One would suppose this writer had gone back to the time when a committee reported to the New Jersey Society, "That after examining among a large number of specimens on the market we find little difference in them. They are all coarse in texture, rough in surface and brittle in substance. They shrink in hardening, blacken in use and are unreliable in duration."

Now, we thought to ourself, if this is so, I will see what time and patience and skill can do to improve it. For two years we worked at it, till we found that by using a proper proportion of gold, platina, silver and tin, these defects were overcome. We gave out samples during the second year to the best and most thoughtful dentists of the State, and at the end of that year we had the pleasure of having that same association pass a resolution of commendation. There are few who do not know of its success.—*Items of Interest.*

I think we cannot be too careful in avoiding lengthy and painful operations for children. I rarely introduce gold fillings, and never very large ones, into the teeth of young children. I refer to the permanent teeth, of course; yet I have seen many temporary teeth filled with gold. Sixth-year molars are sometimes filled with gold soon after eruption, but in such cases they are liable to give out in a short time. A lady in my office, one day, for whose child I was then operating, said to me that the child's eldest sister had previously quite a number of teeth filled by an excellent dentist. He did some very heroic work for her, and was anxious to have all the work done in a very limited time. After the operation was over, the child had St. Vitus dance, and was for a long time in a very nervous condition. —FRANCIS C. E.)

---

COMBINATION OF CEMENT WITH AMALGAM.—Mix the Amalgam in the usual manner, avoiding an excess of mercury, whereas the cement is mixed perhaps slightly thinner than usual; the two are then thoroughly incorporated by means of a stiff spatula. The amount of amalgam used is, in bulk, about one-third to one-half that of the cement. The material may also be prepared by simply dropping the amalgam already mixed into the liquid of the phosphate cement, and then incorporating enough powder to make a stiff paste. The filling is inserted in the same manner as a simple cement filling.—*Cosmos.*

---

TO CLARIFY WAX.—Melt in hot-water bath; then remove from water bath and bring to a slow boil on the stove. Into the boiling wax break a fresh egg, and stir three or four minutes till the egg is thoroughly cooked. Strain through a piece of cheese cloth, to remove all pieces of egg, and you will have your wax as clean and pure as when brought from the dental depot.—*Items of Interest.*

PLASTER OF PARIS FORMULA.—1. To Make Plaster set Hard—Mix best plaster of Paris with about 10 per cent. (more or less, according to effect ascertained by preliminary experiment) of very finely powdered marble (calcium carbonate). Or add to it about 6 per cent. of powdered alum, or about the same amount of ammonium chloride, before mixing with water.

2. To Make Plaster Set Slower—Mix it with 2 to 4 per cent. of powdered altæa root before adding the water. This not only retards the hardening of the plaster, but also enables it to be cut, filed, sawed and turned.

An addition of 8 per cent. of althæa, powder retards the complete setting of the plaster for about one hour, so that the mass can be used for any purpose where it is to remain plastic at least during a portion of that time.—*Amer. Drug.*

---

As an illustration of how happily things dovetail and fit into one another in this world, vegetanarianism comes along as a new fashion, just as Sir James Crichton-Browne, who is out on a physiological hunting expedition, has made the wonderful discovery that we are to lose our positions as carnivora. At the present rates of estimate the time is calculable when we will have no teeth. Out of 1.861 children under twelve years that passed under his critical inspection, only 104 had normal teeth, needing neither filling nor extracting. Various temporizing expedients are suggested, such as school inspection of teeth, for which there will doubtless be a municipal dentist. The outlook is surely awfully awful, excepting to those registered as "mechanical" dentists.

---

Dr. G. T. Phillips, of Rutland, Vt., says common stove blacking, of the consistance of cream, covering the packing of Whitney vulcanizers, makes the joint very tight. Our own experiences has showed this to be good, and there are probably many with whom this will not be new. Some, however, will be thankful for the information.

For years I have not filled the permanent teeth with gold, and it is the one thing that we should impress upon our patients, especially the parents. They say, "Are not these the permanent teeth?" Yes, but gold is not the thing to fill them with. All of the teeth of my patients who have been treated in this manner are better preserved. We all know how much more exhausting it is to fill teeth for children than for adults.—STOCTON (C. E.), *International Dental Journal.*

---

A NICKEL REMOVING SOLUTION.—In order to remove a coating of nickel which does not adhere well M. P. Dronier, in *La Metallurgie*, recommends that the article should be plunged in an oxidizing liquid composed of bichromate of potash, sulphuric acid and water in the proportions ordinarily used for batteries. The article should then be taken out more or less quickly according to the thickness of the deposit and washed and, if necessary, repolished.—*Eng. and Min. Jour.*

---

THERE are all manner of excuses made for the use of tobacco. We now hear it said that its use as a "chaw" keeps the teeth clean. We have been a dentist a good many years, and never saw a clean-mouthed tobacco chewer, though we have seen the mouths of many who did not use it beautifully clean. It is true, as a mopp it does, after a manner, clean the outer surfaces of the teeth, but it leaves the internal surfaces so nasty and full of the stinking stuff, they had much better have used a piece of their wife's mop-rag.

